# Epidemiological investigation of a confirmed case of West Nile virus in Campo Novo do Parecis, Mato Grosso, Brazil, 2025–2026

**DOI:** 10.1590/1980-549720260029

**Published:** 2026-07-27

**Authors:** João Pedro Rocha Nogueira, Cecilia Cintra dos Reis, Alba Valeria Gomes de Melo, Janaina Pauli, Ruberlei Godinho de Oliveira

**Affiliations:** I Secretaria de Estado de Saúde de Mato Grosso – Cuiabá (MT), Brazil.; II Hospital Universitário Júlio Müller – Cuiabá (MT), Brazil.

**Keywords:** West Nile virus, Epidemiological surveillance, Arboviruses, Reverse Transcriptase Polymerase Chain Reaction, Public health

## Abstract

**Objective:**

To describe the epidemiological investigation following laboratory confirmation of West Nile virus in an infant residing in Mato Grosso, Brazil.

**Methods:**

Descriptive study conducted between February 11 and 13, 2026, following notification and diagnostic confirmation by molecular biology. Passive and active surveillance were implemented.

**Results:**

The index case had symptom onset on December 18, 2025, confirmed by molecular biology in a cerebrospinal fluid sample collected on December 20, 2025. In the active investigation, 112 residents were interviewed, identifying 29 symptomatic individuals (25.9%), of whom eight reported neurological manifestations. Environmental conditions favorable to vector proliferation were observed.

**Conclusion:**

The findings suggest possible viral circulation and reinforce the need to intensify integrated human-animal-environmental surveillance, with expanded laboratory and entomological investigation in our territory.

## INTRODUCTION

The West Nile virus (WNV) is an arbovirus of the genus Flavivirus, transmitted mainly by mosquitoes of the genus Culex, with birds serving as natural amplifying reservoirs. Humans and equids are considered accidental and terminal hosts. Human infection is predominantly asymptomatic, although about 20% of those infected develop a nonspecific febrile syndrome and less than 1% progress to neuroinvasive disease, manifesting as meningitis, encephalitis, or acute flaccid paralysis^1^.

WNV was first identified in Brazil in 2009, with serological and molecular evidence in humans, birds, and equids. The first confirmed human case occurred in 2014, in the state of Piauí. Between 2014 and 2024, approximately 110 human cases were recorded across different locations nationwide, suggesting wide geographic dispersion. Recent studies demonstrate active circulation of the virus, including detection in patients with febrile and neurological syndromes, with positivity rates of up to 12.1% in specific investigations. Environmental factors, such as the presence of water bodies, birds, and favorable climatic conditions, contribute to the maintenance of the viral cycle^1,2^.

In Brazil, the occurrence of human cases of West Nile Fever (WNF) is considered an event of epidemiological importance, requiring timely investigation and coordination between epidemiological, environmental, and laboratory surveillance^1^. The confirmation of the first case of WNF in an infant in Mato Grosso, on February 6, 2026, prompted a field investigation aimed at identifying possible additional cases among contacts, as well as characterizing the local epidemiological scenario and assessing environmental factors associated with transmission risk. Despite evidence of viral circulation in Brazil, there is a scarcity of detailed descriptions of investigations with comprehensive field methodologies following the confirmation of human cases. The occurrence of human cases is considered an event of public health relevance, requiring timely investigation and coordination of health surveillance actions^3^.

This is the first confirmed case of WNF in the state of Mato Grosso, constituting an unprecedented event for state surveillance and requiring an immediate response from health teams. Due to the epidemiological importance of this case, this study aimed to describe the course from the onset of symptoms of the index case to the diagnosis and the investigation actions carried out by the Mato Grosso State Health Department in partnership with the Municipal Health Department and the Ministry of Health.

## METHODS

This is a descriptive cross-sectional study with a territorial basis and epidemiological investigation conducted between February 11 and 13, 2026, following the notification of a confirmed case of WNF in the Notifiable Diseases Information System (SINAN) in Campo Novo do Parecis, a municipality in the Southwest Region of Mato Grosso.

### Laboratory confirmation

The index case was a female infant residing in a peri-urban area of Campo Novo do Parecis, with symptom onset on 18/12/2025 and neurological manifestations that led to hospitalization. The first cerebrospinal fluid sample was collected on December 20, 2025 and received at the laboratory on January 19, 2026.

The diagnosis was confirmed by molecular biology through reverse transcription polymerase chain reaction (real-time RT-PCR), with a detectable result for WNV. The reactions were performed in duplicate, with no disagreement between replicates, according to the protocol of the Arbovirology Laboratory of the Evandro Chagas Institute.

The first case of WNF in Mato Grosso considered the contributions and rapid responses, but also the novelty of this case in our territory, with criteria for detection and the absence of clinical suspicion during the hospitalization of the index case. Gaps in the capacity to recognize emerging arboviruses are evident, especially in scenarios where the virus is not considered endemic.

Based on the symptoms presented by the patient, the initial diagnosis was concluded as meningitis, status epilepticus, and difficult-to-control epilepsy, with meningitis being ruled out by laboratory criteria on 22/12/2025, through culture and RT-PCR of cerebrospinal fluid by the Central Laboratory of Mato Grosso (LACEN). There was no suspicion of WNF during the hospitalization period, which occurred between 19/12/2025 and 09/02/2026, and therapeutic management was conducted considering the neurological manifestations, with emphasis on seizure episodes.

After receipt of the cerebrospinal fluid sample, and following the negative results for meningitis, dengue, Chikungunya, and Zika, LACEN forwarded the cerebrospinal fluid sample to the Evandro Chagas Institute (IEC), which expanded the investigation to other arboviruses, including WNV, which showed a reactive result, confirming the unprecedented case in Mato Grosso.

The extended time between the collection of the cerebrospinal fluid (20/12/2025) and the receipt of the sample by IEC (19/01/2026) was due to the time taken by LACEN to carry out investigations of endemic pathogens in Mato Grosso. After obtaining negative results, the sample was forwarded to the reference laboratory (IEC) to expand the laboratory investigation. Until this case was recorded, LACEN did not perform investigations for WNV.

After the case was confirmed, the Health Department of the State of Mato Grosso, with support from the Ministry of Health, deployed a multidisciplinary technical team to conduct a situational assessment, provide guidance on health surveillance actions for the municipality, and begin investigations. An epidemiological survey was carried out, considering the vector behavior ofthe disease and the possibility of vertical transmission, and based on the collected data, a serological survey was subsequently initiated in humans, birds, and vectors.

The Ministry of Health communicated the case to the State Health Department of Mato Grosso, based on the result detectable through RT-PCR for WNF, issued by the IEC. After confirmation, the case was entered into the Notifiable Diseases Information System (Sinan), being the first recorded human case in Mato Grosso. The Ministry of Health is monitoring the entire investigation process and the actions being carried out.

### Epidemiological investigation

Two complementary strategies were adopted:

Passive surveillance: a retrospective search was carried out in the municipal health system for the period from 01/10/2025 to 11/02/2026. Clinical filters compatible with WNF were used, including fever, headache, myalgia, arthralgia, rash, nausea, asthenia, and neurological manifestations (neck stiffness, seizures, altered level of consciousness) to screen for possible infection with WNV.

Active surveillance: territorial delimitation was carried out considering environmental and vector-related characteristics, with home visits within a 10-block radius of the index case’s residence. A structured script was applied to identify symptomatic individuals and collect environmental information, including the presence of breeding sites, domestic animals, birds, and proximity to water bodies. There was direct participation of epidemiological surveillance professionals, environmental surveillance staff, community health agents, and endemic disease control agents in all stages of the field investigation.

The study area corresponded to the neighborhood of residence of the index case, with delimitation based on the dispersal capacity of mosquitoes of the genus *Culex*. This study territory is a planned grid-style development with relatively standardized blocks, comprising approximately 42 blocks. The delimitation was carried out considering the flight range of mosquitoes of the genus *Culex*, the main vectors of WNV, totaling approximately 23.8% of the neighborhood’s territory. Community health agents and vector control agents participated in the investigations, serving as essential actors in the passive and active surveillance process.

Considering the active investigation method, a structured interview script was used containing identification information, contact characterization, clinical investigation, general symptoms, neurological symptoms, exposure, risk conditions, and referrals. The script was developed by the author, based on the West Nile Fever notification form from DATASUS/Ministry of Health. For data organization, the scripts were numbered with questions from 1 to 112.

The *Quantum Geographic Information System software* (version 2.18.26) was used for the spatial description of the index case in Mato Grosso.

#### Data availability statement

All the data set supporting the results of this study was published in the article itself.

## RESULTS

**Figure 1:**
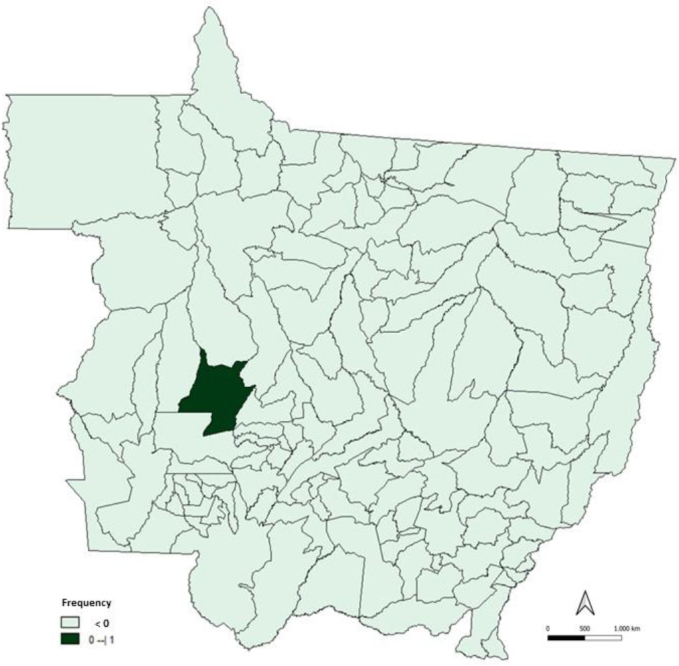
Spatial distribution of the first case of West Nile Fever in Campo Novo do Parecis, Mato Grosso, Brazil, 2025–2026.

The index case, an infant residing in a peri-urban area of the municipality of Campo Novo do Parecis (Figure 1), presented onset of symptoms on December 18, 2025, with neurological manifestations and need for hospitalization. Laboratory confirmation was obtained by RT-PCR in a cerebrospinal fluid sample collected on December 20, 2025, with a detectable result (cycle threshold=35.9; cutoff≤38), performed in duplicate without disagreement. The event was characterized as the first confirmed case of WNF in the state of Mato Grosso.

In the active investigation, conducted within a 10-block radius of the case’s residence, 112 residents were interviewed. Twenty-nine symptomatic individuals were identified, corresponding to 25.9% of those interviewed. The symptomatic individuals were distributed across different age groups, including children, adults, and older adults. Among the 29 symptomatic individuals, 8 (27.6%) reported neurological manifestations, such as neck stiffness, muscle weakness, or excessive drowsiness (Table 1). The remaining individuals presented an unspecific clinical picture, mainly characterized by headache, myalgia, severe fatigue, nausea, diarrhea, and eye pain. Some of the individuals reported having sought medical care, with no record of specific etiological investigation for WNV during the evaluated period (Chart 1).

**Table 1:** Sociodemographic, clinical, and environmental exposure profile of symptomatic individuals identified in active surveillance (n=29). Campo Novo do Parecis, Mato Grosso, 2026.

Variables	n	%
Sex		
Female	21	72.4
Male	8	27.6
Age range		
< 1 year	1	3.4
1 to 14 years	6	20.7
15 to 59 years old	21	72.4
≥ 60 years	1	3.4
General symptoms		
Headache	18	62.1
Myalgia	14	48.3
Nausea and/or vomiting	10	34.5
Intense fatigue	8	27.6
Diarrhea	7	24.1
Retro-orbital pain	7	24.1
Flu-like syndrome (runny nose/cough)	3	10.3
Neurological manifestations		
Muscle weakness	9	31
Neck stiffness	2	6.9
Excessive sleepiness	2	6.9
Environmental exposure		
Proximity to vacant lots	14	48.3
Home-based poultry farming	13	44.8
Presence of stagnant water	10	34.5
Report of mosquito bites	4	13.8
Exhibition in a rural area	3	10.3

**Chart 1:** Data obtained through active investigation in the area of residence of the first confirmed case of West Nile Fever in Campo Novo do Pareci, Mato Grosso, 2025-2026

	Nº	Address	Sex	Age	Is (onset of symptoms)	General symptoms	Neurological symptoms	Medical care	Environment
1	15	Curió 889	f	67	-	-	Stiff neck	No	Mosquito bite; vacant lot; poultry farming; sparrows in the ceiling.
2	17	Arara Azul 689	f	12	-	Headache	-	-	Mosquito bite; vacant lot
3	18	Urapuru 1830	f	59		Headache; muscle pain	-	-	Stagnant water; vacant lot; poultry farming
4	19	Arara Azul 689	f	5	-	Headache	-	-	Wasteland
5	20	Arara Azul 689	m	36	Jan.	Flu-like syndrome and headache	-	-	Wasteland
6	21	Arara Azul 689	f	15	Fev.	Headache	-	-	Wasteland
7	22	Arara Azul 689	f	36	Fev.	Headache	-	-	Wasteland
8	29	Beija Flor 715	m	1	-	-	-	yes	Poultry farming
9	30	Beija Flor 715	f	41	-	Headache; muscle pain; nausea; vomiting and eye pain	-	yes	Stagnant water; Wasteland; Poultry farming
10	31	Beija Flor 715	f	19	05/Fev.	Headache and nausea	-	05/02/2026	Stagnant water; Wasteland; Poultry farming
11	32	Beija Flor 715	f	5	25/Dec.	Headache and nausea	Muscle weakness	25/12/2025	Stagnant water; Wasteland; Poultry farming
12	33	Beija Flor 715	f	40	-	Headache; intense fatigue; nausea; vomiting and diarrhea.	-	yes	Stagnant water; Wasteland; Poultry farming
13	34	-	f	27	-	Headache; muscle pain; intense fatigue; diarrhea and eye pain	Excessive sleepiness; muscle weakness muscular	-	Poultry farming
14	35	Beija Flor 610	f	20	-	Headache; muscle pain; intense fatigue; nausea and vomiting	Stiff neck; Muscle weakness	-	Stagnant water
15	36	Garças 1902	f	15	08/Fev.	Muscle pain; intense fatigue; nausea and chills.	-	-	Symptoms after a walk in a rural area
16	37	Garças 1902	f	35	08/Fev.	Headache; muscle pain; intense fatigue; nausea; vomiting; diarrhea and eye pain	Excessive sleepiness; muscle weakness muscular	-	Symptoms after a walk in a rural area (river). Reports contact with dead birds.
17	38	Ceará 554	f	49	-	Intense fatigue; nausea, vomiting; diarrhea	-	27 and 28/12/2025	Mosquito bite; Poultry farming and horses; residence in a rural area
18	39	Ceará 554	m	6	-	Eye pain	-	yes	-
19	40	Juriti 1734	f	29	-	Flu-like syndrome	-	-	Stagnant water, Poultry farming
20	52	Garças 1787	f	42	Oct.	Headache; muscle pain; intense fatigue; nausea; diarrhea; rash; eye pain	Muscle weakness	yes	Case neighbor; stagnant water; Wastelands; Poultry farming
21	61	Beija Flor 769	m	6 months	-	Runny nose	-	-	-
22	62	Beija Flor 769	m	28	-	Headache; runny nose	-	-	Wasteland
23	75	Juriti 2525	f	55	-	Muscle pain	-	-	-
24	90	Canarinho 950	f	2	-	Muscle pain; nausea; vomiting; diarrhea; runny nose and cough.	Muscle weakness	yes	Mosquito bite; Wasteland
25	92	Garças 105	f	19	-	Headache; muscle pain	Muscle weakness	yes	Stagnant water; Poultry farming
26	93	Juriti 1933	m	43	-	Muscle pain; headache and eye pain	Muscle weakness	-	-
27	101	Papagaio121	f	50	17/Jan.	Headache; muscle pain	-	-	-
28	103	João de Barro 1646	m	41	-	Muscle pain; intense fatigue; diarrhea and eye pain	-	yes	-
29	105	João de Barro 1726	m	38	-	Muscle pain	Muscle weakness	-	Stagnant water

(-): unreported information; f: female; m: male.

Source: Authors, 2026.

The territorial analysis indicated the distribution of symptomatic individuals at different points within the defined area, including residences near the index case’s household. No reports of group travel or a common event outside the investigated territory were identified. At the hospital where the patient first received care, healthcare professionals who had direct clinical contact with the case were identified. One professional reported a self-limited febrile episode in the subsequent weeks, consistent with the expanded clinical definition adopted in the investigation.

The environmental assessment showed frequent presence of containers with standing water in the external areas of residences, vacant lots with accumulated waste, and inadequate drainage (Table 1). Domestic poultry farming was observed on several properties, as well as pigs in adjacent areas. The region is close to rural areas and natural water bodies. Residents reported a high density of mosquitoes, especially during the nighttime period (Chart 1).

## DISCUSSION

The molecular confirmation of this case of WNF in a female infant, recorded in Mato Grosso, reinforces the hypothesis of silent circulation of the virus in the territory, already indicated by studies in sentinel animals such as horses and birds, which play a key role in viral amplification, as well as the need for syndromic surveillance in our territory. The occurrence in infants raises additional concerns due to the immaturity of the immune system and the possibility of atypical neurological clinical progression, which indicates the consolidation of the virus in transitional biomes in Brazil^4,5^.

Evidence of viral circulation in multiple states, including the North, Northeast, and Central-West regions, points to a pattern of spread possibly associated with ecological and environmental factors. The transmission dynamics of WNV involve an enzootic cycle between birds and mosquitoes of the genus Culex, with humans acting as incidental hosts^1^. The identification of 29 symptomatic individuals in the municipality of Campo Novo do Pareci, including individuals with neurological manifestations, raises the hypothesis of possible silent circulation of WNV, evidenced by the diagnostic window of this case, which prevents vector control and transmission-blocking measures, allowing the virus to become established in the local enzootic cycle^6^.

Although there is still no additional laboratory confirmation from the contacts and the infant’s mother, the clinical and environmental context suggests a favorable scenario for transmission. The presence of potential breeding sites and the raising of birds in the household environment may contribute to the maintenance of the enzootic cycle of WNV^4^. The interface between peri-urban and rural areas increases the complexity of surveillance, requiring integration between human, animal, and environmental components^5^.

The first confirmed case of WNV in Mato Grosso in an infant reflects the importance of intersectoral communication within health services. The coordination among care, surveillance, and laboratory services is essential for the speed of responses to unprecedented events^2^. The expansion of laboratory testing, in addition to the endemic pathogens of Mato Grosso, allowed the detection of the case with the collaboration of IEC. New laboratory studies are being conducted in order to understand the epidemiological impact of WNV in the municipality of Campo Novo do Parecis, considering the human-animal-environment characteristics of the condition.

The identification of this case demonstrates the importance of active surveillance of arboviruses, illustrating the risk of transmission and outbreaks due to the synanthropic characteristics related to arthropod vectors. Environmental characteristics must also be considered. When analyzed together with the natural history of WNF, it can be inferred that Mato Grosso, located in the Central-West region, is considered an ecological corridor for migratory birds, specifically the Pantanal region, with the presence of arbovirus vectors in all municipalities of the state. Syndromic surveillance actions in relation to other non-endemic arboviruses in Mato Grosso highlight the importance of research and the possible underreporting of WNV in humans in Brazilian territory^6^. The *One Health* concept illustrates the unprecedented response to this case of WNF, being an integrated approach that recognizes and acts on the inseparable interconnection between human, animal, and environmental health^7^.

The detection of WNV associated with the identification of symptomatic cases and the presence of environmental risk factors indicates the need to intensify epidemiological, entomological, and laboratory surveillance in health services. WNF presents a higher frequency of severe clinical manifestations in elderly and immunocompromised individuals, with cases in infants rarely described in the literature, which highlights a possible additional risk factor for WNV transmissibility in the region, as well as the proximity of risk involving the territorial base of the municipality of this first WNF case, along with the report of the first case of WNV in animals in the Pantanal^8,9,10^. Although it is considered of low incidence in humans in the country, WNV shows consistent evidence of silent circulation in Brazil, with potential for expansion, through the strengthening of integrated human-animal-environmental surveillance and the expansion of diagnostic capacity essential for early detection and prevention of severe cases^11^. The climatic conditions in Mato Grosso, with a hot and dry climate, may have negative impacts on vector abundance and mosquito viral dynamics in our territory^12^. It is also noteworthy that clinical overlap with other arboviruses may hinder differential diagnosis, making the use of serological and molecular laboratory methods essential in the investigation of non-endemic arboviruses in Mato Grosso^2^.

Among the limitations, the absence of laboratory confirmation in symptomatic individuals identified with possible underdiagnosis stands out, as well as the reliance on self-reported information in household interviews. However, the findings are consistent with the scenario described in recent literature and contribute to the advancement of knowledge about the epidemiology of WNV in Brazil.

In summary, the findings of this study reinforce the importance of conducting serological surveys, entomological monitoring, and strengthening timely diagnostic capacity, with an emphasis on differential diagnosis among endemic arboviruses in Mato Grosso. The need for intersectoral and interdisciplinary actions coordinated among health services is highlighted, aiming at the early detection of WNV circulation.
