# Supplementary material for: Epidemiological investigation of a confirmed case of West Nile virus in Campo Novo do Parecis, Mato Grosso, Brazil, 2025–2026
Source: Rev Bras Epidemiol. 2026 Jul 27;29:e260029. doi: 10.1590/1980-549720260029 (PMC13440505; doi:10.1590/1980-549720260029)
Supplement: Translation

# Translation: Investigação epidemiológica de caso confirmado de vírus do Nilo Ocidental em Campo Novo do Parecis, Mato Grosso, Brasil, 2025–2026

**DOI:** 10.1590/1980-549720260029.2

**Authors:** João Pedro Rocha Nogueira, Cecilia Cintra dos Reis, Alba Valeria Gomes de Melo, Janaina Pauli, Ruberlei Godinho de Oliveira

**Affiliations:** I Secretaria de Estado de Saúde de Mato Grosso – Cuiabá (MT), Brasil.; II Hospital Universitário Júlio Müller – Cuiabá (MT), Brasil.

**Keywords:** Vírus do Nilo Ocidental, Vigilância epidemiológica, Arboviroses, Reação em Cadeia da Polimerase Via Transcriptase Reversa, Saúde pública

## Abstract

**Objetivo:**

Descrever a investigação epidemiológica após confirmação laboratorial de vírus do Nilo Ocidental em lactente residente em Mato Grosso, Brasil.

**Métodos:**

Estudo descritivo conduzido entre 11 e 13 de fevereiro de 2026, após notificação e confirmação diagnóstica por biologia molecular. Foram implementadas vigilância passiva e vigilância ativa.

**Resultados:**

O caso-índice apresentou início dos sintomas em 18 de dezembro de 2025, confirmado por biologia molecular em amostra de líquor coletada em 20 de dezembro de 2025. Na investigação ativa, 112 moradores foram entrevistados, identificando-se 29 sintomáticos (25,9%), dos quais oito relataram manifestações neurológicas. Foram observadas condições ambientais favoráveis à proliferação vetorial.

**Conclusão:**

Os achados sugerem possível circulação viral e reforçam a necessidade de intensificação da vigilância integrada humano-animal-ambiental, com ampliação da investigação laboratorial e entomológica em nosso território.

## INTRODUÇÃO

O vírus do Nilo Ocidental (VNO) é um arbovírus do gênero *Flavivírus*, transmitido principalmente por mosquitos do gênero *Culex*, tendo aves como reservatórios amplificadores naturais. Humanos e equídeos são considerados hospedeiros acidentais e terminais. A infecção humana é predominantemente assintomática, embora cerca de 20% dos infectados desenvolvam síndrome febril inespecífica e menos de 1% evoluam para doença neuroinvasiva, manifestando-se como meningite, encefalite ou paralisia flácida aguda^1^.

O VNO foi identificado no Brasil pela primeira vez em 2009, com evidências sorológicas e moleculares em humanos, aves e equídeos. O primeiro caso humano confirmado ocorreu em 2014, no estado do Piauí. Entre 2014 e 2024, foram registrados aproximadamente 110 casos humanos distribuídos em diferentes localidades nacionais, sugerindo ampla dispersão geográfica. Estudos recentes demonstram circulação ativa do vírus, incluindo detecção em pacientes com síndrome febril e neurológica, com positividade de até 12,1% em investigações específicas. Fatores ambientais, como presença de coleções hídricas, aves e condições climáticas favoráveis, contribuem para a manutenção do ciclo viral^1,2^.

No Brasil, a ocorrência de casos humanos da Febre do Nilo Ocidental (FNO) é considerada evento de importância epidemiológica, demandando investigação oportuna e articulação entre vigilância epidemiológica, ambiental e laboratorial^1^. A confirmação do primeiro caso de FNO em uma lactente em Mato Grosso, em 06 de fevereiro de 2026, motivou a realização de investigação de campo com o objetivo de identificar possíveis casos adicionais entre os contatos, bem como a caracterização do cenário epidemiológico local, e avaliar fatores ambientais associados ao risco de transmissão. Apesar de evidências de circulação viral no Brasil, há escassez de descrições detalhadas de investigações com metodologias detalhadas de campo após confirmação de casos humanos. A ocorrência de casos humanos é considerada evento de relevância em saúde pública, exigindo investigação oportuna e articulação das ações de vigilância em saúde^3^.

Trata-se do primeiro caso confirmado de FNO no estado de Mato Grosso, configurando evento inédito para a vigilância estadual e demandando resposta imediata das equipes de saúde. Devido à importância epidemiológica deste caso, este estudo pretende descrever o percurso entre o início de sintomas do caso-índice, até o diagnóstico e as ações de investigações executadas pela Secretaria de Estado de Saúde de Mato Grosso em parceria com a Secretaria Municipal e o Ministério da Saúde.

## MÉTODOS

Trata-se de estudo transversal descritivo de base territorial com investigação epidemiológica realizada entre 11 e 13 de fevereiro de 2026, após notificação de caso confirmado de FNO no Sistema de Informação de Agravos de Notificação (SINAN) em Campo Novo do Parecis, município da Região Sudoeste de Mato Grosso.

### Confirmação laboratorial

O caso-índice foi um lactente do sexo feminino, residente em área periurbana de Campo Novo do Parecis, com início dos sintomas em 18/12/2025 e manifestações neurológicas que motivaram hospitalização. A primeira amostra de líquor foi coletada em 20 de dezembro de 2025 e recebida no laboratório em 19 de janeiro de 2026.

O diagnóstico foi confirmado por biologia molecular por meio da reação em cadeia da polimerase via transcriptase reversa (RT-PCR em tempo real), com resultado detectável do VNO. As reações foram realizadas em duplicata, sem discordância entre replicatas, conforme protocolo do Laboratório de Arbovirologia do Instituto Evandro Chagas.

O primeiro caso de FNO no Mato Grosso considerou as contribuições e as respostas rápidas, mas também o ineditismo desse caso em nosso território, com critérios para a detecção e a ausência de suspeição clínica durante a internação do caso-índice. Evidenciam-se lacunas na capacidade de reconhecimento de arboviroses emergentes, especialmente em cenários onde o vírus não é considerado endêmico.

A partir da sintomatologia apresentada pela paciente, o diagnóstico inicial foi fechado em meningite, mal convulsivo e epilepsia de difícil controle, sendo meningite descartada por critério laboratorial em 22/12/2025, por meio de cultura e RT-PCR em líquor pelo Laboratório Central de Mato Grosso (LACEN). Não houve suspeição de FNO durante o período de internação, que ocorreu entre 19/12/2025 e 09/02/2026, e o manejo terapêutico foi conduzido considerando as manifestações neurológicas, com destaque para as crises convulsivas. Após recebimento da amostra de líquor, e a partir do resultado negativo para meningites, dengue, Chikungunya e Zika, o LACEN encaminhou a amostra de líquor para o Instituto Evandro Chagas (IEC), que ampliou a pesquisa para outros arbovírus, incluindo VNO, que apresentou resultado reagente, confirmando o caso inédito em Mato Grosso.

O tempo prolongado entre a coleta do líquor (20/12/2025) até o recebimento da amostra pelo IEC (19/01/2026) se deu devido ao tempo em que o LACEN levou para realizar as pesquisas de patógenos endêmicos em Mato Grosso, e a partir dos resultados negativos, encaminhou-se a amostra ao laboratório de referência (IEC) para ampliar a investigação laboratorial. Até o registro desse caso, o LACEN não realizava pesquisas de VNO.

Após a confirmação do caso, a Secretaria do Estado de Saúde de Mato Grosso, com o apoio do Ministério da Saúde, deslocou uma equipe técnica multiprofissional para a realização do diagnóstico situacional, orientações das ações de vigilância em saúde para o município e início das investigações. Foi realizado um levantamento epidemiológico, considerando o comportamento vetorial da doença e a possibilidade de transmissão vertical, e, a partir dos dados levantados, com posterior início do inquérito sorológico em humanos, aves e vetores.

O Ministério da Saúde comunicou o caso à Secretaria do Estado de Saúde de Mato Grosso, a partir do resultado detectável por meio de RT-PCR para FNO, emitido pelo IEC. Após confirmação, o caso foi inserido no Sistema de Informação de Agravos de Notificação (Sinan), sendo o primeiro caso registrado em humano em Mato Grosso. O Ministério da Saúde acompanha todo o processo de investigação e as ações executadas.

### Investigação epidemiológica

Foram adotadas duas estratégias complementares:

Vigilância passiva: realizou-se busca retrospectiva no sistema municipal de saúde no período de 01/10/2025 a 11/02/2026. Utilizaram-se filtros clínicos compatíveis com FNO, incluindo febre, cefaleia, mialgia, artralgia, exantema, náuseas, astenia e manifestações neurológicas (rigidez de nuca, convulsões, alterações do nível de consciência) para rastreio de possível infecção pelo VNO.

Vigilância ativa: realizou-se delimitação territorial considerando características ambientais e vetoriais, com visitas domiciliares em raio de 10 quarteirões da residência do caso-índice. Aplicou-se roteiro estruturado para identificação de sintomáticos e levantamento de informações ambientais, incluindo presença de criadouros, animais domésticos, aves e proximidade com coleções hídricas. Houve participação direta de profissionais de vigilância epidemiológica, vigilância ambiental, agentes comunitários de saúde e agentes de combate a endemias em todas as etapas da pesquisa de campo.

A área de estudo correspondeu ao bairro de residência do caso-índice, com delimitação baseada na capacidade de dispersão de mosquitos do gênero *Culex*. Esse território do estudo é um loteamento planejado em grade com quadras relativamente padronizadas, com aproximadamente 42 quadras. A delimitação se deu considerando a capacidade de voo dos mosquitos do gênero *Culex*, principais vetores do VNO, totalizando aproximadamente 23,8% do território do bairro. Os agentes comunitários de saúde e os agentes de combate às endemias participaram das investigações, sendo atores imprescindíveis no processo de vigilância passiva e ativa.

Considerando o método de investigação ativa, utilizouse um roteiro de entrevista estruturado contendo informações de identificação, caracterização de contato, investigação clínica, sintomas gerais, sintomas neurológicos, exposição, condições de risco e encaminhamentos. O roteiro foi elaborado pelo autor, baseado na ficha de notificação de Febre do Nilo Ocidental do DATASUS/Ministério da Saúde. Para a organização dos dados, os roteiros foram enumerados com perguntas de 1 a 112.

Utilizou-se o *software Quantum Geographic Information System* (versão 2.18.26) para a descrição espacial do caso-índice em Mato Grosso.

#### Declaração de Disponibilidade de Dados

Todo o conjunto de dados que dá suporte aos resultados deste estudo foi publicado no próprio artigo.

## RESULTADOS

O caso-índice, de lactente residente em área periurbana do município de Campo Novo do Parecis (Figura 1), apresentou início dos sintomas em 18 de dezembro de 2025, com manifestações neurológicas e necessidade de hospitalização. A confirmação laboratorial ocorreu por RT-PCR em amostra de líquor coletada em 20 de dezembro de 2025, com resultado detectável (ciclo limiar=35,9; ponto de corte≤38), realizado em duplicata sem discordância. O evento foi caracterizado como o primeiro caso confirmado de FNO no estado de Mato Grosso.

**Figura 1:** Distribuição espacial do primeiro caso de Febre do Nilo Ocidental em Campo Novo do Parecis, Mato Grosso, Brasil, 2025–2026.

Na investigação ativa, realizada em raio de 10 quarteirões da residência do caso, foram entrevistados 112 moradores. Identificaram-se 29 indivíduos sintomáticos, correspondendo a 25,9% dos entrevistados. Os sintomáticos estavam distribuídos em diferentes faixas etárias, incluindo crianças, adultos e idosos. Entre os 29 sintomáticos, 8 (27,6%) relataram manifestações neurológicas, como rigidez de nuca, fraqueza muscular ou sonolência excessiva (Tabela 1). Os demais apresentaram quadro clínico inespecífico, caracterizado principalmente por cefaleia, mialgia, fadiga intensa, náuseas, diarreia e dor ocular. Parte dos indivíduos referiu ter procurado atendimento médico, sem registro de investigação etiológica específica para VNO no período avaliado (Quadro 1).

**Tabela 1:** Perfil sociodemográfico, clínico e de exposição ambiental dos sintomáticos identificados na investigação ativa (n=29). Campo Novo do Parecis, Mato Grosso, 2026

Variáveis	n	%
Sexo		
Feminino	21	72,4
Masculino	8	27,6
Faixa etária		
< 1 ano	1	3,4
1 a 14 anos	6	20,7
15 a 59 anos	21	72,4
≥ 60 anos	1	3,4
Sintomas gerais		
Cefaleia	18	62,1
Mialgia	14	48,3
Náuseas e/ou vômitos	10	34,5
Fadiga intensa	8	27,6
Diarreia	7	24,1
Dor retro-ocular	7	24,1
Síndrome gripal (coriza/tosse)	3	10,3
Manifestações neurológicas		
Fraqueza muscular	9	31
Rigidez de nuca	2	6,9
Sonolência excessiva	2	6,9
Exposição ambiental		
Proximidade com terrenos baldios	14	48,3
Criação domiciliar de aves	13	44,8
Presença de água parada	10	34,5
Relato de picadas de mosquitos	4	13,8
Exposição em área rural	3	10,3

**Quadro 1:** Dados obtidos por meio da investigação ativa no território de residência do primeiro caso confirmado de Febre do Nilo Ocidental em Campo Novo do Pareci, Mato Grosso, 2025-2026

	Nº	Endereço	Sexo	Idade	Is (início dos sintomas)	Sintomas gerais	Sintomas neurológicos	Atendimento médico	Ambiente
1	15	Curió 889	f	67	-	-	Rigidez na nuca	Não	Picada de mosquito; terreno baldio, criação de aves; pardais no forro
2	17	Arara Azul 689	f	12	-	Cefaleia	-	-	Picada de mosquito; terreno baldio
3	18	Urapuru 1830	f	59		Cefaleia; mialgia	-	-	Água parada; terreno baldio; criação de aves
4	19	Arara Azul 689	f	5	-	Cefaleia	-	-	Terreno baldio
5	20	Arara Azul 698	m	36	jan	Síndrome gripal e cefaleia	-	-	Terreno baldio
6	21	Arara Azul 689	f	15	fev	Cefaleia	-	-	Terreno baldio
7	22	Arara Azul 689	f	36	fev	Cefaleia	-	-	Terreno baldio
8	29	Beija Flor 715	m	1	-	-	-	sim	Criação de aves
9	30	Beija Flor 715	f	41	-	Cefaleia; mialgia; náusea; vômito e dor ocular	-	sim	Água parada; terreno baldio; criação de aves
10	31	Beija Flor 715	f	19	05/fev	Cefaleia e náusea	-	05/02/2026	Água parada; terreno baldio; criação de aves
11	32	Beija Flor 715	f	5	25/dez	Cefaleia e náusea	Fraqueza muscular	25/12/2025	Água parada; terreno baldio; criação de aves
12	33	Beija Flor 715	f	40	-	Cefaleia; fadiga intensa; náusea; vômitos e diarreia	-	sim	Água parada; terreno baldio; criação de aves
13	34	-	f	27	-	Cefaleia; mialgia; fadiga intensa; diarreia e dor ocular	Sonolência excessiva; fraqueza muscular	-	Criação de aves
14	35	Beija Flor 610	f	20	-	Cefaleia; mialgia; fadiga intensa; náusea e vômito	Rigidez na nuca; fraqueza muscular	-	Água parada
15	36	Garças 1902	f	15	08/fev	Mialgia; fadiga intensa; náusea e calafrio	-	-	Sintomas após passeio em área rural
16	37	Garças 1902	f	35	08/fev	Cefaleia; mialgia; fadiga intensa; náusea; vômito; diarreia e dor ocular	Sonolência excessiva; fraqueza muscular	-	Sintomas após passeio em área rural (rio). Relata contato com aves mortas
17	38	Ceará 554	f	49	-	Fadiga intensa; náusea, vômitos; diarreia	-	27 e 28/12/2025	Picada de mosquito; criação de aves e equinos; residência em área rural
18	39	Ceará 554	m	6	-	Dor ocular	-	sim	-
19	40	Juriti 1734	f	29	-	Síndrome gripal	-	-	Água parada, criação de aves
20	52	Garças 1787	f	42	out	Cefaleia; mialgia; fadiga intensa; náuseas; diarreia, exantema; dor ocular	Fraqueza muscular	sim	Vizinho do caso; água parada; terreno baldio; criação de aves
21	61	Beija Flor 769	m	6 meses	-	Coriza	-	-	-
22	62	Beja Flor 769	m	28	-	Cefaleia; coriza	-	-	Terreno baldio
23	75	Juriti 2525	f	55	-	Mialgia	-	-	-
24	90	Canarinho 950	f	2	-	Mialgia; náusea; vômito; diarreia; coriza e tosse	Fraqueza muscular	sim	Picada de mosquito; terreno baldio
25	92	Garças 105	f	19	-	Cefaleia; mialgia	Fraqueza muscular	sim	Água parada; criação de aves
26	93	Juriti 1933	m	43	-	Mialgia; cefaleia e dor ocular	Fraqueza muscular	-	-
27	101	Papagaio 121	f	50	17/jan	Cefaleia; mialgia	-	-	-
28	103	João de Barro 1646	m	41	-	Mialgia; fadiga intensa; diarreia e dor ocular	-	sim	-
29	105	João de Barro 1726	m	38	-	Mialgia	Fraqueza muscular	-	Água parada

(-): informações não relatadas; f: feminino; m: masculino.

Fonte: Autores, 2026.

A análise territorial indicou distribuição dos sintomáticos em diferentes pontos da área delimitada, incluindo residências próximas ao domicílio do caso-índice. Não foi identificado relato coletivo de viagem ou evento comum fora do território investigado. No hospital onde ocorreu o primeiro atendimento da paciente foram identificados profissionais que tiveram contato assistencial direto com o caso. Um profissional relatou episódio febril autolimitado nas semanas subsequentes, compatível com definição clínica ampliada adotada na investigação.

A avaliação ambiental evidenciou presença frequente de recipientes com água parada nas áreas externas das residências, terrenos baldios com acúmulo de resíduos e drenagem inadequada (Tabela 1). Observou-se criação domiciliar de aves em diversas propriedades, além de suínos em áreas adjacentes. A região apresenta proximidade com áreas rurais e coleções hídricas naturais. Os moradores relataram elevada densidade de mosquitos, especialmente no período noturno (Quadro 1).

## DISCUSSÃO

A confirmação molecular desse caso de FNO em uma lactente do sexo feminino, registrado em Mato Grosso, reforça a hipótese de circulação silenciosa do vírus no território, já apontada por estudos em animais sentinelas, como equinos e aves, que desempenham papel fundamental na amplificação viral, bem como da necessidade de vigilância sindrômica em nosso território. A ocorrência em lactentes suscita preocupações adicionais devido à imaturidade do sistema imunológico e à possibilidade de evolução clínica neurológica atípica que indica a consolidação do vírus em biomas de transição no Brasil^4,5^.

A evidência de circulação viral em múltiplos estados, incluindo as regiões Norte, Nordeste e Centro-Oeste, aponta para um padrão de disseminação possivelmente associado a fatores ecológicos e ambientais. A dinâmica de transmissão do VNO envolve um ciclo enzoótico entre aves e mosquitos do gênero *Culex*, com humanos atuando como hospedeiros incidentais^1^. A identificação de 29 sintomáticos no município de Campo Novo do Pareci, incluindo indivíduos com manifestações neurológicas, levanta a hipótese de possível circulação silenciosa do VNO, evidenciada pela janela diagnóstica desse caso que impede medidas de controle vetorial e bloqueio de transmissão, permitindo que o vírus se estabeleça no ciclo enzoótico local^6^.

Embora ainda não haja confirmação laboratorial adicional dos contatos e da mãe da lactente, o contexto clínico e ambiental sugere cenário favorável à transmissão. A presença de criadouros potenciais e a criação de aves no ambiente domiciliar podem contribuir para a manutenção do ciclo enzoótico do VNO^4^. A interface entre áreas periurbanas e rurais amplia a complexidade da vigilância, exigindo integração entre os componentes humano, animal e ambiental^5^.

O primeiro caso de FNO confirmado em Mato Grosso em uma lactente reflete, ainda, a importância da comunicação intersetorial nos serviços de saúde. A interlocução dos serviços de atenção, vigilância e laboratório é fundamental para a agilidade em respostas a eventos inéditos^2^. A ampliação da pesquisa laboratorial, além dos patógenos endêmicos de Mato Grosso, permitiu a detecção do caso com a colaboração do IEC. Novas pesquisas laboratoriais estão sendo realizadas a fim de compreender o impacto epidemiológico do VNO no município de Campo Novo do Parecis, considerando as características humano-animal-ambiental do agravo.

A identificação desse caso demonstra a importância da vigilância ativa frente às arboviroses, ilustrando o risco de transmissões e surtos devido às características sinantrópicas relativas aos vetores artrópodes. As características ambientais também devem ser consideradas. Quando analisadas em conjunto com a história natural da FNO, infere-se que o Mato Grosso, por localizar-se na região Centro-Oeste, é considerado um corredor ecológico de aves migratórias, especificamente a região do Pantanal, com a presença de vetores das arboviroses em todos os municípios do estado. As ações de vigilância sindrômica em relação às outras arboviroses não endêmicas em Mato Grosso mostram a importância da pesquisa e a possível subnotificação do VNO em humanos em nosso território^6^. O conceito de Saúde Única (*One Health*) ilustra a resposta inédita desse caso de FNO, sendo uma abordagem integrada que reconhece e atua sobre a interconexão inseparável entre saúde humana, animal e ambiental^7^.

A detecção do VNO associado à identificação de sintomáticos e à presença de fatores ambientais de risco indica a necessidade de intensificação da vigilância epidemiológica, entomológica e laboratorial nos serviços de saúde. A FNO apresenta maior frequência de manifestações clínicas graves em idosos e em indivíduos imunocomprometidos, sendo os casos em lactentes raramente descritos na literatura, o que evidencia um possível fator de risco adicional da transmissibilidade do VNO na região, bem como a proximidade do risco envolvendo a base territorial do município desse primeiro caso de FNO, com o relato do primeiro caso de VNO em animais no Pantanal^8,9,10^. Embora seja considerada de baixa incidência no país em humanos, a VNO apresenta evidências consistentes de circulação silenciosa no Brasil, com potencial de expansão, por meio do fortalecimento da vigilância integrada humano-animal-ambiental e da ampliação da capacidade diagnóstica essencial para detecção precoce e prevenção de casos graves^11^. As condições climáticas em Mato Grosso, com clima quente e seco, podem ter impactos negativos na abundância de vetores e na dinâmica viral do mosquito em nosso território^12^. Ressalta-se, ainda, que a sobreposição clínica com outras arboviroses pode dificultar o diagnóstico diferencial, tornando essencial o uso de métodos laboratoriais sorológicos e moleculares na pesquisa de arbovírus não endêmicos em Mato Grosso^2^.

Entre as limitações, destacam-se a ausência de confirmação laboratorial nos sintomáticos identificados com possíveis subdiagnóstico e a dependência de informações autorreferidas nas entrevistas domiciliares. No entanto, os achados são consistentes com o cenário descrito na literatura recente e contribuem para o avanço do conhecimento sobre a epidemiologia do VNO no país.

Em síntese, os achados deste estudo reforçam a importância da realização de inquéritos sorológicos, do monitoramento entomológico e do fortalecimento da capacidade diagnóstica oportuna, com ênfase no diagnóstico diferencial entre arboviroses endêmicas em Mato Grosso. Destaca-se a necessidade de ações intersetoriais e interdisciplinares articuladas entre os serviços de saúde, visando à detecção precoce da circulação do VNO.

## COMITÊ DE ÉTICA:

Trata-se de investigação epidemiológica realizada no âmbito das ações de vigilância em saúde, conforme atribuições institucionais da Secretaria de Estado de Saúde, dispensada de apreciação por Comitê de Ética em Pesquisa, conforme normativas nacionais vigentes.
